# Born Perfect

**Published:** 1871-05

**Authors:** 


### BORN PERFECT.

"Has it any deformity?" is one of the first questions the mother wishes answered about her new-born babe. It should be a source of daily and life-long thankfulness, deep, and sincere, and humble, that a child is not deaf, or dumb, or blind, or hump-backed, or club-footed, or hare-lipped, or dreadfully marked on the face, a lunatic or a fool. The time will come when child-bearing will become more a matter of scientific calculation and arrangement than it now is. If the same care was exercised in this direction as in the management of the domestic animals, it certainly would elevate the race in physical beauty, perfection, and power; would expand the intellect and mould the heart, the moral emotions, to a pattern nearer to the Infinite.

A city paper announces that "The two-headed girl are on exhibition in Philadelphia. They is eighteen years of age, and waltzes together beautifully. She are twin sisters." Nathaniel Wilson died lately in Boston at the age of thirty years, weighing seventy pounds; the flesh seemed plastered on the bones; his arms measured two and a half inches round, and his legs were not much larger; and for six years he had been in this sad condition; and it is significantly added, "He seems to have inherited from his mother the extraordinary peculiarities which marked his physical condition." The question here arises before the intelligent mind, Is it not a great wrong, an inhumanity, for any person to marry, who is not in vigorous health? If we shrink from marrying into an insane family, a like prudence applies to diseased connections.

